# A Systematic Review of Clinical Psychophysiology of Obsessive–Compulsive Disorders: Does the Obsession with Diet Also Alter the Autonomic Imbalance of Orthorexic Patients?

**DOI:** 10.3390/nu15030755

**Published:** 2023-02-02

**Authors:** Carlo Pruneti, Gabriella Coscioni, Sara Guidotti

**Affiliations:** 1 Clinical Psychology, Clinical Psychophysiology and Clinical Neuropsychology Laboratory, Department of Medicine and Surgery, University of Parma, 43126 Parma, Italy; 2 Graduate School of Psychology, Boston College, Newton, MA 02467, USA

**Keywords:** nutrition, diet, orthorexia, obsessive–compulsive disorder, autonomic imbalance, psychophysiology, psychophysical health, mental health, heart rate variability, autonomic nervous system

## Abstract

(1) Background: A new mental illness is attracting the attention of researchers and mental health professionals. Orthorexia nervosa (ON) is a possible new mental disorder, the main symptom of which is an obsessive and insecure focus on healthy foods and consequent compulsive behaviors. There is a common consensus among researchers that ON is considered partly overlapping with obsessive–compulsive disorders (OCDs). (2) Methods: MEDLINE and Scopus were searched for articles published in the last 10 years regarding the psychophysiological aspects of OCD and ON. Eight studies met the eligibility criteria. The inclusion criteria encompassed adults diagnosed with OCD and/or ON. However, only studies involving OCD patients were found. (3) Results: Some research groups have shown that OCD disorders can be considered among anxiety disorders because they are characterized by anxious hyper activation. Other research, however, has shown profiles characterized by low psychophysiological reactivity to stressful stimuli. Despite this, there seems to be a consensus on the poor inhibition abilities, even when activation is low, and the dissociation between cognitive and psychophysiological activation emerged. (4) Conclusions: However discordant, some points seem to bring the researchers to agreement. In fact, there is consensus on conducting a multidimensional assessment that can measure all of the aspects of suffering (cognition, emotion, and behavior) and highlight the poor body–mind integration. This clinical approach would make it possible to propose interventions aimed at treating some mental illnesses such as food obsession that can paradoxically impair the psychophysical balance. Nevertheless, the applied systematizing approach to existing studies on ON is very much needed for better understanding of the psychophysical nature of this new mental illness and its implications for prevention and treatment.

## 1. Introduction

Recently, the scientific community has studied a new clinical condition. Despite the few studies available, the term orthorexia has already entered the vocabulary of clinical professionals. This term refers to the self-imposed dietary rules designed to promote health. However, as early as 1997, Steven Bratman [1] pointed out that the pursuit of “extreme dietary purity” due to an exaggerated focus on food can lead to some forms of eating disorders (EDs) having harmful and counterproductive consequences [2,3]. Orthorexia is a neologism coined from Greek that can be directly translated into “proper appetite” [4]. In contrast, orthorexia nervosa (ON) is an expression created to refer to a possible new ED, the main symptom of which is an obsessive and insecure focus on eating foods considered healthy [5]. Although this disorder has not yet been inserted by the diagnostic manuals, the literature review conducted by Cena and colleagues [5] highlighted two main pieces of criteria that could become pathognomonic: (1) obsessive focus (inflexible dietary rules, recurrent and persistent food-related worries, and compulsive behaviors) on dietary practices that are believed to promote optimal well-being; and (2) consequent clinically significant impairment (i.e., medical or psychological complications, great distress, and/or impairment of important areas of functioning) [6]. 

As to eating behavior, the same authors [5] identified different levels of quality of food. More specifically, sometimes healthy/proper/correct/safe characteristics of food emerge, while at other times the definition of food considers specific aspects such as organic, biologically pure, or related to food production. In other cases, the definition of healthy food seems not to refer to the biological quality but to pseudo-moral aspects. Therefore, the diet followed by subjects with ON is characterized by a restrictive scheme in which the distortion of the eating habits and the avoidance of specific foods can lead to the shortage of essential nutrients or malnutrition and underweight [7]. Nevertheless, specific behavioral aspects can also be found in terms of ritualized and/or strictly controlled eating habits [5,6,7]. 

There is an ongoing debate in the literature as to whether ON should be considered just an impaired eating habit, a disorder falling under EDs or obsessive–compulsive disorders (OCDs), or even a disorder in its own right [4,8,9,10]. However, it seems that the most frequently used terms among researchers to describe ON are the following: obsession, fixation, and worry/concern [10]. Obsession indicates a persistent and disturbing thought, while fixation is a stereotyped behavior related to an obsessive and unhealthy preoccupation. Concern refers to an uneasy state of blended interest, uncertainty, and apprehension, while preoccupation may be considered a synonym of concern with a higher degree of alarm representing the state someone is in when giving all of his/her attention to something [5]. The association between these psychological variables was well described by Barthels and colleagues in a 2021 study [11]. These authors demonstrated that higher levels of healthy habits, perceptions of autonomic sensations, hypochondriacal worry, and absorption can be found in individuals with symptoms of orthorexia. In other words, these results highlighted that there are some characteristic features of illness anxiety and dysfunctional cognition typical of somatic symptom disorders. 

Precisely with regard to the theme of corporality, ON can be considered as the cause or the effect of mental disorders [12,13]. Indeed, obsessive attention to nutrition is sometimes consequential to organic conditions that require lifestyle changes [7,14,15]. Furthermore, the cognitive schemes associated with a predisposition to obsessiveness favor the adoption of restrictive and/or rigid diets such as those of the ON [7]. Moreover, as frequently happens in the field of neurotic disorders, there are some comorbidities [16], including somatic symptom disorders or hypochondriasis (when concerns about health are disproportionate), anxiety and depressive disorders (frequently associated with OCD), and full-blown ED (i.e., severe anorexic conditions) [4,9,10] underpinned by rigid personality structure including perfectionism and obsessive–compulsive traits [16,17] as well as negative affectivity and psychoticism [16]. Thus, the ON syndrome could be another case of OCD in which the pathological control of eating could reflect the existence of a mental condition that compromises the psychophysical balance [3].

In light of the emerging data from the researchers’ analysis of ON, an overview of OCD seems necessary to better understand this condition.

OCD is characterized by highly disturbing, intrusive, and unwanted thoughts or obsessions, which the patient unsuccessfully tries to regulate by engaging in senseless rituals or avoiding stimuli that triggers obsessions [18]. The propensity to perform compulsive behaviors despite negative consequences is considered the prominent feature of the OCD. For this reason, OCD was conceptualized as a disorder of self-control and behavioral inhibition [19,20]. More specifically, the neurocognitive perspective of the disorder focuses on the diminished capacity to inhibit intrusive cognition and stop a repetitive activity. The impairment in inhibitory regulation is considered as a risk for emotional dysregulation typical in psychopathological disorders [21,22].

In addition, it has been demonstrated that individuals with OCD have difficulty shifting between mental processes to generate adaptive behavioral responses, especially in the context of their symptoms. In support of this, several neurocognitive studies suggest that cognitive flexibility (i.e., attentional set shifting, alternation, reversal learning, and cognitive control) assessed through the Trail Making Test (B version) and the Stroop Test is impaired in individuals with OCD [23.27]. The researchers agree that under-activation of prefrontal regions, especially the orbitofrontal cortex (OFC), may be responsible for the functional alterations described [23,24,25,26,27,28,29]. All of these studies support a neurobiological model of OCD, suggesting an important role for dysfunctional loops in cortico-striato-thalamo-cortical (CSTC) circuits [20,30] as well as the involvement of limbic structures linked to the etiology of the disease [31,32,33,34,35].

Since 2000, Thayer and Lane [36] have suggested that there might be a strong interconnection between cognitive functioning and psychophysiological arousal in OCD. More specifically, these authors developed a model that suggests that some variations in cardiovascular functioning are part of a network, which integrates autonomic, attentional, and emotional systems. Thayer and Lane [36] found that diminished heart rate variability (HRV) corresponds to an impairment in cognitive inhibition in healthy subjects [22,37]. Moreover, HRV reflects the beat-to-beat changes in heart rate (HR) that result from the interplay between sympathetic and parasympathetic nervous system influences on the heart. New perspectives in clinical psychophysiology are currently underlying physiological mechanisms and properties of the ultra-low frequency (ULF), very low frequency (VLF), low frequency (LF), and high frequency (HF) bands. There is a common consensus among researchers that the power in the LF band can be influenced by vagal, sympathetic, and baroreflex mechanisms depending on the context, whereas HF power is produced by the efferent vagal mechanism due to respiratory activity. It is often assumed that a low LF/HF ratio reflects greater parasympathetic activity relative to sympathetic activity. In contrast, a high LF/HF ratio may indicate higher sympathetic activity [10]. Although both branches influence HR, evidence suggests that the parasympathetic influence is dominant [38,39,40], indicating that the heart is under the tonic inhibitory control peripherally via the vagus nerve. The vagal modulation of cardiac activity allows the organism to be quickly activated in order to respond to the environmental challenges with flexibility and resilience. Moreover, there are direct and indirect pathways by which the prefrontal cortex modulates parasympathetic activity via subcortical circuits [41]. Thayer and Lane [42] demonstrated that an inhibitory cortical–subcortical circuit supports physiological, affective, and cognitive regulation. Moreover, the activity of this neural circuit can be vagally mediated. According to this model, higher levels of HRV (i.e., greater vagal tone) at rest are a product of a system in which the prefrontal cortex exerts inhibitory control over subcortical circuits, thus allowing the organism to respond to environmental challenges in a controlled and adaptive manner when needed. Indeed, a number of studies found that individual differences in resting HRV might be indicative of the self-regulatory capacity. For instance, individuals with high HRV at rest show enhanced performance on executive function tasks including those that assess working memory [43], attention [44], and motor response control [45]. By contrast, those with low HRV are characterized by self-regulatory deficits including poor attentional control, ineffective emotion regulation, and behavioral inflexibility [46,47]. Importantly, individual differences in HRV can be observed during resting baseline periods and appear to be relatively stable over time [48]. Despite these associations, only a few studies investigated the role of resting HRV in the context of control over unwanted thoughts and the use of thought suppression in specific psychopathological disorders, such as OCD. In this regard, Ingjaldsson, Laberg, and Thayer [49] found that people with higher resting HRV rely less on thought suppression and experience fewer intrusive thoughts. In addition, individuals who engage in chronic thought suppression often report high levels of depression, anxiety, and psychological stress [50]. In another more recent study, Gillie, Vasey, and Thayer [51] investigated whether individual differences in resting HRV were associated with the occurrence of unwanted thoughts and the ability to control such intrusions via thought suppression. This study was based on the idea that the occasional occurrence of such thoughts is a common experience even in non-clinical samples, but they are still experienced as recurrent, distressing, and uncontrollable [52]. Moreover, attempts to control intrusions using strategies including thought suppression are often unsuccessful and may serve to paradoxically increase their frequency [53,54]. In addition, some researchers demonstrated that the availability of cognitive resources as indexed by working memory capacity and inhibitory control can facilitate thought suppression success [55,56] presumably by influencing the efficiency of the effortful operating process that works to allocate attention to distractor thoughts [57]. Given that HRV has been linked to both working memory capacity and inhibition more generally [58], those with higher levels of HRV, when instructed to engage in thought suppression, may experience a greater decline in intrusive thoughts than individuals with lower HRV because they are able to allocate their attention elsewhere [51]. Regarding patients suffering from OCD, the consensus among researchers is that diminished physiological flexibility [59] connects to the central nervous system (CNS) in a constant hyper-aroused state [60,61].

Therefore, according to some researchers, OCD would partly overlap with the criteria of anxiety disorders, at least from a neuropsychological and psychophysiological point of view. In both conditions, the deficit in inhibitory processes is considered as a risk for emotional regulation and psychopathological manifestation [62]. More specifically, the rigid emotional response style that does not reflect the environmental demands characterizes the anxiety disorders and is associated with the inability to inhibit inappropriately anxious responses in non-threatening situations. However, other research has also described an opposite trend. Some researchers observed that the resting HRV values of OCDs do not differ from those of healthy controls [63].

For these reasons, the purpose of this review is to analyze the studies present in the literature on the psychophysiology of OCD spectrum disorders including ON. In addition, considering that the diagnostic manuals prior to the DSM-5 included OCD among anxiety disorders, research that investigated the psychophysical alteration in multiple psychopathological categories was included. Furthermore, it is hypothesized that an investigation of autonomic imbalance of ON can be performed by including it within the OCD spectrum. Although this new clinical condition is not yet included among the diagnostic manuals, the opinion of several researchers [5,11] seems to converge into the belief that obsession, fixation, and preoccupation are the pathognomonic criteria that will most likely allow it to be included among OCDs.

## 2. Materials and Methods

### 2.1. Data Source/Literature Search 

This systematic review was conducted in accordance with the Preferred Reporting Items for Systematic Reviews and Meta-Analyses (PRISMA) Statement. MEDLINE and Scopus were searched for articles published in the last 10 years. The following search terms were used: (Psychophysiology OR Autonomic Imbalance OR Heart Rate Variability OR skin conductance OR electro dermal activity OR galvanic skin response OR muscle tension OR electromyography) AND (Orthorexia OR Obsessive-Compulsive Disorder). 

Two reviewers independently evaluated publications for inclusion, based on their titles and abstracts. Full texts were then retrieved for those articles deemed eligible and considered for inclusion independently by both reviewers.

### 2.2. Inclusion and Exclusion Criteria 

Studies were included in the systematic review if they: (1) examined a sample of adult (≥18 years of age) patients; (2) quantitatively investigated the psychophysiology and the autonomic imbalance in OCD and ON patients where obsessive–compulsive symptomatology was measured by a recognized scale (i.e., Anxiety Disorders Interview Schedule-IV (ADIS-IV); Minnesota Multiphasic Personality Inventory 2 (MMPI-2); Obsessive Compulsive Inventory—Revised (OCI-R); Structured Clinical Interview for DSM-IV axis 1 disorders (SCID-I); Yale–Brown Obsessive–Compulsive Scale (Y-BOCS)); (3) were observational, quasi-experimental, or experimental studies; (4) were published in peer-reviewed journals or in books and book chapters in any language; and (5) measured obsessions and compulsions with valid self-report measures. Studies were excluded if they (1) were qualitative, reviews, meta-analyses, case studies, theses, dissertations, or conference presentations; (2) did not specifically measure obsessions and compulsions; (3) enrolled patients with multiple diseases without performing subgroup analyses for psychopathological patients. 

### 2.3. Data Extraction 

Data relevant for the review included details about participants, the assessment of obsessions and compulsions, and the association between psychopathological symptoms of OCD/ON and psychophysiological investigations. Additional extracted data included year of publication, sample size, study design (i.e., observational study, quasi experimental, and experimental), and data analysis (e.g., correlation, regression). 

## 3. Results

### 3.1. Search Results

**Figure 1:**
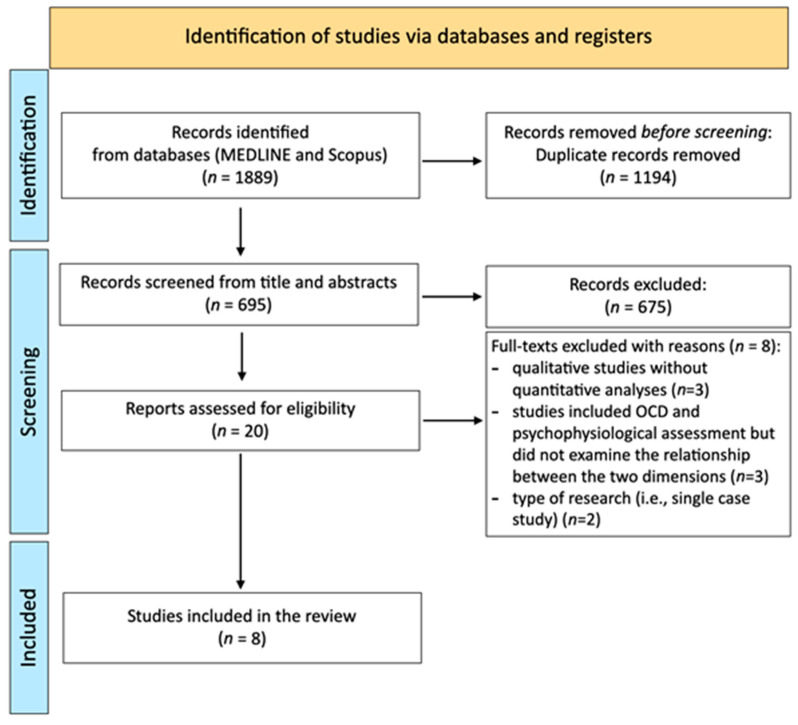
PRISMA flow diagram showing the selection of primary studies.

The initial search resulted in 1889 studies. Of these, 1869 were eliminated through the initial screening process. Of the remaining 20 studies, 8 were not considered due to type of research (e.g., single case study, *n* = 2), absence of quantitative analyses (*n* = 3), or absence of the analysis between OCD symptoms and psychophysiological assessment (*n* = 3). Overall, eight studies were included in the review. In Figure 1 the outcomes of the study selection process are presented. 

### 3.2. Characteristics of Included Studies 

Details of included studies are shown in Table 1. Two studies had a cross-sectional design and involved only patients diagnosed with OCD [64,65], while two other cross-sectional studies also involved other patients suffering from other psychopathological disorders (i.e., PD, SAD, GAD, MDE, and AN) (in accordance with the DSM-IV or DSM-5 criteria) [66,67]. Two of these studies also had a case-control design [65,66], and one of these was an fMRI study as well [65]. Three other studies had a case-control design [61,68,69], and one of these was also quasi-experimental [49]. Lastly, an observational and longitudinal (3 (group) × 6 (stimulus) repeated measures) study was also included [70]. 

### 3.3. Results for Obsessive–Compulsive Disorder 

Considering the last decade, one of the most relevant studies for the purpose of this review is by Havnen et al. [64]. This research group involved 31 patients aged between 22 and 54 years old diagnosed with OCD in accordance with the Structured Clinical Interview (SCID) for DSM-IV axis 1 disorders [71]. The study analyzed the relationship between the obsessive symptoms, the performance obtained at the Stroop Test, and the autonomic imbalance in terms of HRV values. The results showed that the frequency band of the HRV that reflects the activity of the parasympathetic branch of the autonomic nervous system (ANS) (the HF-HRV) negatively correlates with the scores of the Yale–Brown Obsessive–Compulsive Scale (Y-BOCS) (*r* = −0.319, *p* = 0.04) that detects obsessions and compulsions and the scores of the Stroop Test (*r* = −0.427, *p* = 0.02). These authors confirmed for the first time the relationship between executive functions (cognitive inhibition) and emotional arousal in a group of patients with OCD. More specifically, it seems that the psychophysiological parameter of the HRV is modulated by the activity of cortical frontal areas. 

In the same year, a study by Pittig et al. [68] involved a larger sample size made up of 131 participants, including 82 treatment-seeking patients with a primary DSM-IV [72] anxiety disorder and 39 non-anxious controls. Participants were diagnosed with a DSM-IV criteria for panic disorder (PD) (with or without agoraphobia), social anxiety disorder (SAD), generalized anxiety disorder (GAD), or OCD with a clinical severity rating (CSR) of four or higher. The most relevant result consists of the evidence that control subjects report significantly higher baseline HF-HRV than all the patients with anxiety disorders combined. Considering the relaxation phase, there were no significant differences in HR change between all of the patients, while significantly lower HF-HRV during the first 30 s of the hyperventilation phase was found in all anxiety disorders patients combined, including OCD patients. The data demonstrated that OCD patients report a similar pattern of HR and HRV, responding like the other anxiety disorders.

A more recent study by Olbrich and colleagues [61] points in this direction. These authors involved 51 unmedicated OCD patients (subsequently treated with cognitive behavioral therapy (CBT), *n* = 18; selective serotonin-reuptake inhibitors (SSRIs), *n* = 11; or a combination, *n* = 22) and 28 healthy controls. A 15-minute resting state electrocardiogram (ECG) was recorded in order to assess the function of the ANS by using the HRV. The main results showed a significantly higher HR in the unmedicated OCD-sample in comparison to the controls (*F* = 8.62, *p* < 0.001). Moreover, it has emerged that a significantly larger HF power can differentiate a group of responders to the therapy from a group of non-responders (*F* = 4.98, *p* = 0.03). This research group concluded that in OCD patients there seems to be an overactivation of sympathetic activity and a decrease in parasympathetic activity. 

Opposite results were described in a clinical study conducted by Pruneti and colleagues [66] in which 89 subjects diagnosed with OCD, GAD, PD, major depressive episode (MDE), or anorexia nervosa (AN) were involved. The control group consisted of 34 healthy subjects. Various psychophysiological parameters were registered, including surface electromyography (sEMG), skin conductance level and response (SCL/SCR), peripheral temperature (PT), and HR, and compared between the groups of patients selected. Every physiological parameter was recorded in three different phases: baseline, stress (patients were subjected to a mental arithmetic task), and recovery. In contrast to previous studies, these authors described lower levels of HR in OCD patients. The comparison between groups showed lower levels close to significance of HR in the stress phase compared to the control group (*U* = 124, *p* = 0.06) and in all of the three phases of the registration compared with the GAD (*U* = 79, *p* = 0.00, *U* = 94.5, *p* = 0.00, *U* = 83, *p* = 0.00, respectively). On the contrary, HR levels of OCD patients were very similar to the values of AN and MDE groups. Considering other parameters besides HR, OCD reported significantly lower levels of SCR in all of the three phases of the psychophysiological assessment in comparison to GAD (*U* = 51, *p* = 0.00, *U* = 52, *p* = 0.00, *U* = 55.5, *p* = 0.00, respectively), PD (*U* = 16, *p* = 0.00, *U* = 18, *p* = 0.00, *U* = 15, *p* = 0.00, respectively), and the controls (*U* = 113, *p* = 0.03, *U* = 111, *p* = 0.03, *U* = 116, *p* = 0.04, respectively). Looking at the sEMG values at rest and during the stress session, it has emerged that frontal muscle tension is the only parameter that did not show significant statistical differences between control and pathological groups because all of the patients, including OCD, reported higher levels compared to the healthy controls. These authors concluded that the autonomic imbalance of OCD patients is characterized by a general neurovegetative hypoactivation.

Similar results regarding the skin conductance were also described two years earlier by the same authors [67] which detected significant differences between OCD and anxious patients (GAD and PD) in the three phases of the recording of the psychophysiological profile (baseline, stress, and recovery) (GAD vs. OCD: *U* = 110, *p* < 0.001, *U* = 26, *p* < 0.001, *U* = 95, *p* < 0.001; PD vs. OCD: *U* = 83, *p* < 0.001, *U* = 84, *p* < 0.001, *U* = 94, *p* < 0.001). In addition, Pruneti et al. noted that the SCR values in OCD patients seem to fall within the typical range (2–6 µS) in all of the phases of psychophysiological recording even if other interesting aspects emerge. More specifically, it seems that slight reactivity to stress is still present (baseline vs. stress condition: *W* = 96, *p* < 0.05) and characterized by the inability to relax after the stress (stress condition vs. recovery: *W* = 141, *p* = n.s.).

Another study also examined the trend of the SCR parameter. A study by Witton et al. [70] compared 25 OCD patients with 21 subjects diagnosed with non-OCD anxiety disorders and 25 healthy participants with respect to trait, self-reported, facial sEMG, and GSR disgust responses in order to analyze the level of psychophysiological activation connected to the emotion of disgust. This research revealed that individuals with OCD showed greater disgust propensity and self-reported disgust toward images of body waste compared to healthy and anxious participants (group x image interaction for self-reported disgust ratings: *F*(10,340) = 4.02, *p* < 0.001). However, there were no group differences in physiological responses. After controlling for trait disgust, obsessive beliefs positively correlated with increased self-reported disgust to neutral images and increased levator labii activity (detected through the sEMG) to negative non-disgusting images (*r* = 0.28, *p* < 0.05).

Furthermore, Milad and colleagues [65] examined the dissociation between different measures in patients diagnosed with OCD. In a 2013 study, the psychophysiological and neurobiological correlates of 21 patients with OCD during an experimental paradigm of conditioned fear extinction were analyzed. The comparison between the group of OCD patients and a group of 21 healthy subjects revealed that individuals with OCD show impaired extinction recall. Furthermore, a dissociation between the two measures emerged. More specifically, while the expression of conditioned fear and its subsequent extinction is intact (as measured by SCR: late vs. early extinction training, *F* = 0.77, *p* = 0.39), an impairment in the recall of the extinction memory emerges in terms of a decreased activation of the ventro-medial prefrontal cortex (vmPFC) across all the experimental phases (extinction training OCD>HC: *t* = −2.98, *p* < 0.05, extinction recall OCD>HC: *t* = −3.28, *p* < 0.05). Moreover, OCD symptom severity was positively correlated with the magnitude of extinction memory recall as well as with functional responses of the vmPFC (*r* = 0.56, *p <* 0.05). 

Lastly, the potential differences between 37 patients with OCD and 56 healthy subjects in a classical fear-conditioning task were investigated by Pöhlchen et al. [69]. These authors analyzed some physiological parameters such as pupil dilation, startle amplitude, and SCR across conditioning and extinction recorded at day 1 while the extinction recall and reinstatement was assessed at day 2. For SCR, no evidence for models including either a group effect or an interaction with group (stimulus × group, time × group, time × stimulus × group) during any task phase was observed. For the startle response, a trend toward higher startle amplitudes during extinction for OCD emerged (*BF*(10) = 0.224, *d* = 0.03).

## 4. Discussion

The literature review revealed noteworthy aspects despite the fact that the results obtained by various researchers are sometimes discordant. In particular, the research conducted by Havnen et al. [64], Pittig et al. [68], and Olbrich et al. [61] showed a general condition of sympathetic activation in patients diagnosed with OCD. The first study precisely described how a mental task requiring the involvement of fronto-executive skills, and in particular the inhibitory control, promotes the maintenance of the physiological hyperactivation in a group of patients with OCD. Moreover, poor parasympathetic activation correlates with obsessive–compulsive symptoms. Similar results were observed by Pittig et al. [68] in the same year. These authors described poor involvement of the parasympathetic branch of the ANS in OCD subjects both at rest and under two different and opposite experimental conditions (relaxation and hyperventilation). According to these authors, the patients’ psychophysiological profile was similar to that of a group of anxious subjects. From these results it may be concluded that OCD is considered in all respects a facet of anxiety disorders. Finally, the study by Olbrich et al. [61] compared OCD subjects with control subjects and showed that even in a 15-min resting recording the patients were hyperactive when considering cardiac function parameters (HR and HRV).

On the other hand, opposite results were described by Pruneti and colleagues in 2014 and 2016 [66,67]. Specifically, in 2016, the authors observed the trend of different psychophysiological parameters (HR, SCL-SCR) as well as frontal muscle tension. Through the performance of a comparison between OCD, PD, GAD, MDE, AN, and controls, previous studies were confirmed [73,74,75,76]. More specifically, it emerged that the profile of OCD was very similar to that of depressed and anorexic patients, while it differed from anxious disorders considering both the HR and the SCR. In contrast, considering the muscle tension, all patients reported significantly different and higher values in comparison with the healthy group. Two years earlier, the same authors considered only the skin conductance parameter and did not involve healthy subjects. A significantly different value between OCD and anxiousness was observed, but other considerations were possible even at a descriptive level. For instance, psychophysiological values of OCDs were within the normal range in all of the three phases of detection (baseline, stress, and recovery), although they differed from controls by an albeit slight reactivity to the stressful stimulus and difficulty in inhibiting psychophysiological activation and recovering the pre-stress level of rest. 

Furthermore, other studies in the literature investigated the psychophysiological activation in patients with OCD in terms of sympathetic indices such as GSR and sEMG. For example, the study conducted by Witton et al. in 2015 [70] found that there are differences between the subjective and objective responses of patients with OCD and controls. The data showed that there was a dissociation between perception and autonomic activation in subjects with OCD in a scientific paradigm that proposed stimuli with body waste images. However, it is not the first time that a dissociation between explicit and implicit measures has emerged [77]. The dissociation between psychophysiological and cognitive aspects was also reported by the research conducted by Milad and colleagues [65] in which the responses of patients with OCD were compared with those of healthy subjects in a fear extinction paradigm. Consistent with theories supporting the inhibition deficit, it was found that some brain areas responsible for inhibitory control remained activated despite the visible recovery at the psychophysiological level. In addition, a similar paradigm was implemented in the study by Pöhlchen et al. [69]. These researchers investigated some components of skin conductance in a fear conditioning task and found that only subtle differences at the level of galvanic response could be detected.

In summary, despite the divergent results and conflicting opinions among researchers, it appears that an autonomic impairment characterizes patients with OCD. The studies of Witton et al. [70], Pittig et al. [68], and Olbrich et al. [61] fully support the theories of the inhibitory deficit because the physiological parameters connected to the cardiac function described a scarce vagal activity. However, some clinical considerations can be observed in the studies of Pruneti et al. [66,67] who carried out a psychophysiological assessment by detecting various physiological parameters. In particular, HR and SCR appear to be significantly lower in patients with OCD than in anxious subjects vs. healthy controls, and their condition is very similar to the hypoactivation typical of depression and severe anorexic syndromes. However, in 2014, the same authors highlighted the slight reactivity to stressful stimuli in patients with OCD and the significant and marked difficulty in relaxing. In other words, this study highlighted the difficulty of inhibiting the sympathetic activation required during stress. Furthermore, the study that investigated the trend of skin conductance in a fear extinction task stressed the need to deepen the dissociation between neural cognition (in terms of poor inhibition of the vmPFC area) and autonomic activity (with fair recovery of conductance) [65].

Basically, all of the studies analyzed agree on the fact that a psychophysiological activation can be observed in patients with OCD, mainly following a cognitive activation. In fact, all the research demonstrated the poor ability to inhibit sympathetic activation following the performance of a mental task. These results therefore support the various theories that explain OCD as a psychopathological disorder where cognitive processes are able to generate an emotional arousal that the individual is unable to inhibit. The data seem to support the commonly shared idea within the clinic that compulsions are the only way to release somatic tension. According to this perspective, the behaviors implemented by OCD would fall within one of the strategies, albeit maladaptive, to manage the distress generated by thoughts. It is probably for this reason that many studies do not detect significant differences compared to healthy people and instead highlight a less marked emotionality compared to patients suffering from anxiety disorders. Moreover, it is possible that the differences noted by the various researchers may be due, at least in part, to the different research paradigms implemented and the psychophysiological parameters detected.

### 4.1. Study Limitations 

The results of the present study should be interpreted in the light of some limitations. First, the inclusion of articles from peer-reviewed journals has caused the exclusion of the gray literature. In addition, only studies involving patients diagnosed with OCD and/or who reported a clinically significant OCD tests score were enclosed. Bearing in mind that obsession, fixation, low-calorie diet, and concern for one’s silhouette can bring together different mental disorders, this limitation may hinder the drawn conclusions. Lastly, the main limitation of the present review is its consideration of OCD and ON together despite the fact that the latter disease is not yet included in diagnostic manuals. This choice was made in light of the recent scientific papers [5,11] that have pointed out the presence of pathognomonic features of ON that are very similar to those of the OCD spectrum. The same researchers claim that future diagnostic manuals will place them within the same psychopathological category. However, this speculation remains the most important limitation of our work. Despite these considerations, our review sheds light on a possible clinically relevant but understudied association between ON and OCD and highlights the need for further studies in this regard. Should the scientific evidence confirm that ON is largely overlapping with OCD, it might be worthwhile to further investigate this paradoxical condition whereby a “healthy” nutrition can even become harmful.

### 4.2. Directions for Future Research 

Our systematic review found a significant gap in the literature because there are no clinical psychophysiology studies of ON and only a few of OCD. Indeed, it is not easy to conduct clinical trials involving patients and carrying out multidimensional assessments. Investigating the cognitive, behavioral, and emotional–psychophysiological dimensions requires sophisticated tools and equipment as well as specialized personnel for the execution and analysis of the data carried out (usually psychologists who are experts in clinical psychophysiology and biofeedback). However, in light of the findings highlighted by this review, the need for a multidimensional assessment that can describe the characteristics of OCD spectrum disorders on all levels has further importance. Future studies will need to consider the various facets of psychological suffering by including psychophysiological responses within the diagnostic evaluation. This approach would fall within the recently proposed research domain criteria (RDoC) for the study of mental disorders, which aim to characterize psychopathology in terms of normal and abnormal biological and behavioral processes rather than as categories of discrete symptoms [78]. As far as the physiological parameters are concerned, it could also be interesting to investigate the various dissociations between the indices recorded. For instance, it might be useful to further investigate HRV (especially HF-HRV) and SCR within the same experimental paradigm because it is not clear why conductance activity is so inhibited. Several studies in the literature demonstrated that SCR reflects the cognitive processing of emotional stimuli in healthy subjects [79,80,81]; therefore, this difference with OCD subjects needs to be investigated.

From a general future perspective, the need to standardize the methods of the psychophysiological research will emerge. This improvement may eliminate the discrepancies that originate from the different procedures implemented by the researchers. 

Lastly, the need to adequately define the ON is also apparent. The classification and categorization in the diagnostic manuals of this psychopathological condition would allow appropriate investigations at the psychophysiological level to be carried out. Future studies are needed to confirm the hypotheses that guided our research in conducting a review on OCD with speculative hypotheses on ON. Obviously, the intent of this review was to shed light on the ON and guide future studies that can confirm our hypotheses.

### 4.3. Clinical Implications 

The current findings certainly need to be further investigated and explored before being generalized to the clinical setting. However, some noteworthy considerations deserve to be mentioned. 

All of the studies have demonstrated a low vagal activity which does not allow recovery of the autonomic imbalance, although some research demonstrated excessive sympathetic activity, while other studies have not. Overall, a first clinical implication leads mental health specialists to adequately evaluate the management of these patients’ emotions because sometimes a clear dysregulation may not emerge. In fact, it is possible that patients with OCD use behavioral manifestations to ease the anxious tension generated by the high activity of their cognitive processes [82]. This mechanism could result in an apparent emotional control that hides the real difficulties in managing internal states.

In addition, the inability to inhibit physiological arousal and to recover autonomic balance even in the absence of a clear sympathetic overactivity has been underlined [66,67]. In any case, this aspect has strong clinical relevance because it highlights the need to treat these patients by facilitating vagal activation through psychological interventions aimed at favoring relaxation. However, further studies are needed before these results can be translated into clinical interventions because the strong involvement of cognitive processes in OCD suggest the use of psychotherapy [16,82,83]. In light of these considerations and of the documented dissociation between cognitive–behavioral and emotional–psychophysiological aspects, clinical research needs further investigations before providing clinical–therapeutic suggestions.

## 5. Conclusions

In summary, the spectrum of OCD is commonly considered within the neuropsychiatric diseases with structural [84] and neurophysiological [60] alterations. This scientific evidence could support the clinical practice of mental health specialists in the diagnostic process. In fact, distinguishing an anxious syndrome from an obsessive syndrome can be very useful for clinicians and for patients considering that very often the onset of symptoms is followed by a long period necessary for the diagnosis and the start of the most appropriate treatment [85]. Therefore, it is even more important to provide patients with a simple and rather short path to diagnosis and treatment initiation, optimally knowing that a patient is more likely to respond to a specific treatment [61].

The intent of our review was partially satisfied by analyzing studies that considered patients with OCD. Unfortunately, not having found research on ON in the literature, it is only possible to hypothesize similarities between the two conditions. However, according to the most recent review that identifies the diagnostic criteria frequently used to identify the ON [5,6,7], the pathognomonic characteristics of OCD seem to have emerged. In particular, obsessiveness and compulsions linked to excessive somatic tension seem to unite the two disorders. For this reason, it was considered useful to investigate the psychophysiology of OCD to study ON as well.

Considering the impact that a mental disorder can have on the body, it is considered important to intercept those mental disorders characterized precisely by a poor mind–body integration [86,87].

## Figures and Tables

**Table 1:** Characteristics of Included Studies.

Authors	Sample	Study Design	Measures	Main Results
Havnen et al. [64]	n = 31 OCD patients	Cross-sectional	*Psychopathological Assessment:* SCID-I; Y-BOCS *Neuropsychological Assessment:* Stroop Test *Physiological Assessment:* HR and HRV	HF-HRV negatively correlates with Y-BOCS and Stroop test.
Olbrich et al. [61]	n = 51 OCD patients n = 28 HC	Case-control and Quasi-experimental	*Psychopathological Assessment:* GCI; Y-BOCS *Physiological Assessment:* HR and HRV	Significantly higher HR in the unmedicated OCD-sample in comparison to the HC. Moreover, HF-HRV Power differentiates responders to non-responders.
Pittig et al. [68]	n = 82 patients with PD, SAD, GAD, or OCD n = 39 HC	Case-control	*Psychopathological Assessment:* ADIS-IV; ASI; PSWQ; Padua Inventory-Revised; FQ-S *Physiological Assessment:* HR and HRV	Significantly higher HF-HRV (baseline) in HC compared with all the patients’ categories. No significant differences in HR (relaxation) change between all anxiety disorders. Significantly lower HF-HRV (hyperventilation phase) in all anxiety disorders compared with HCs.
Pruneti et al. [66]	n = 89 patients with PD, GAD, MDE, AN, or OCD n = 34 HC	Cross-sectional and Case-control	*Psychopathological Assessment:* MMPI-2; SQ; PSQ *Physiological Assessment:* sEMG, SCL/SCR, PT, HR and HRV	Significantly lower levels of HR in OCD patients compared with HC and GAD in all of the three phases of the PSP. Lower levels of SCR in OCDs along with MDE, AN, and HCs, compared with PD and GAD. OCDs, along GAD, PD, MDE, and AN, report significantly higher levels of sEMG compared with HCs.
Pruneti et al. [67]	n = 104 patients with GAD, MDE, PD, or OCD	Cross-sectional	*Psychopathological Assessment:* MMPI-2; SQ; PSQ *Physiological Assessment:* sEMG, SCL/SCR, PT, HR and HRV	Significant differences between OCD and anxious patients (GAD and PD) with regards to the SCR values in the three phases of the PSP. In addition, SCR values in OCD patients fall within the typical range in all of the phases of the PSP. Only a slight reactivity to stress is present but characterized by the inability to relax.
Whitton et al. [70]	n = 25 patients with OCD n = 21 patients non-OCD anxiety disorders n = 25 HC	Observational and Longitudinal	*Psychopathological Assessment:* ADIS-IV; OCI-R; DASS-21; OBQ-44; DPSS-R; DS-R *Physiological Assessment:* facial EMG and EDA	Greater disgust propensity and self-reported disgust to images of body waste in OCDs compared to HC and anxious participants. No group differences in physiological responses. After controlling for trait disgust, obsessive beliefs positively correlates with increased self-reported disgust to neutral images and increased levator labii activity to negative non-disgusting images.
Pöhlchen et al. [69]	n = 37 patients with OCD n = 26 HC	Case-control	*Psychopathological Assessment:* Y- BOCS, OCI-R, HAM-A, HAM-D *Physiological Assessment:* SCR and EMG	No group differences in SCR, pupillometry, or subjective ratings in extinction learning and extinction recall. Only subtle differences in startle responses during extinction (higher amplitudes in OCD patients).
Milad et al. [65]	n = 21 patients with OCD n = 21 HC	Cross-sectional, Case-control, and fMRI study	Psychopathological Assessment: SCID-IV; Y-BOCS Physiological Assessment: SCR Neural Assessment: fMRI BOLD signals	Impaired extinction recall in OCD patients. Regarding the fMRI data, patients with OCD showed significantly reduced activation in the vmPFC across training phases as well as in the caudate and hippocampus during fear conditioning, and in the cerebellum, posterior cingulate cortex, and putamen during extinction recall. In addition, the OCD symptom severity positively correlated with the magnitude of extinction memory recall and the functional responses of the vmPFC while they negatively correlated with the functional responses of the dorsal anterior cingulate cortex.

## Data Availability

The data presented in this study are available upon reasonable request from the corresponding author.

## Abbreviations

ADIS-IV = Anxiety Disorders Interview Schedule-IV; AN = anorexia nervosa; ANS = autonomic nervous system; ASI = Anxiety Sensitivity Index; CBT = cognitive behavioral therapy; CGI = Clinical Global Impression Scale; CNS = central nervous system; CSR = clinical severity rating; CSTC = cortico-striato-thalamo-cortical circuit; DASS-21 = Depression, Anxiety and Stress Scales; DPSS-R = Disgust Propensity and Sensitivity Scale—Revised; DSM = Diagnostic and Statistical Manual of Mental Disorders; DS-R = Disgust Scale—Revised; ECG = electrocardiogram; ED = eating disorder; EDA = electrodermal activity; fMRI = functional magnetic resonance imaging; FQ-S = Fear Questionnaire—Social Phobia Subscale; GAD = generalized anxiety disorder; HAM-A = Hamilton Anxiety Rating Scale; HAM-D = Hamilton Depression Rating Scale; HC = healthy control; HF = high frequency; HR = heart rate; HRV = heart rate variability; LF = low frequency; MDE = major depressive disorder; MMPI-2 = Minnesota Multiphasic Personality Inventory 2; OBQ-44 = Obsessive Beliefs Questionnaire 44; OCD = obsessive–compulsive disorder; OCI-R = Obsessive Compulsive Inventory—Revised; OFC = orbitofrontal cortex; ON = orthorexia nervosa; PRISMA = Preferred Reporting Items for Systematic Reviews and Meta-Analyses; PD = panic disorder; PSP = psychophysiological stress profile; PSQ = P Stress Questionnaire; PT = peripheral temperature; PSWQ = Penn State Worry Questionnaire; RDoC = research domain criteria; SAD = social anxiety disorder; SCID-I = Structured Clinical Interview for DSM-IV axis 1 disorders; SCL/SCR = skin conductance level and response; sEMG = surface electromyography; SQ = symptom questionnaire; SSRI = selective serotonin-reuptake inhibitor; ULF = ultra-low frequency; VLF = very low frequency; vmPFC = ventromedial prefrontal cortex; Y-BOCS = Yale–Brown Obsessive–Compulsive Scale.
